# Ensemble Deep Learning for Cervix Image Selection toward Improving Reliability in Automated Cervical Precancer Screening

**DOI:** 10.3390/diagnostics10070451

**Published:** 2020-07-03

**Authors:** Peng Guo, Zhiyun Xue, Zac Mtema, Karen Yeates, Ophira Ginsburg, Maria Demarco, L. Rodney Long, Mark Schiffman, Sameer Antani

**Affiliations:** 1 Communications Engineering Branch, Lister Hill National Center for Biomedical Communications, U.S. National Library of Medicine, National Institutes of Health, Bethesda, MD 20894, USA; zhiyun.xue@nih.gov (Z.X.); rlong@mail.nih.gov (L.R.L.); santani@mail.nih.gov (S.A.); 2 Ifakara Health Institute, P.O. Box 53 Ifakara, Tanzania; zacmtema@gmail.com; 3 Department of Medicine, Queen’s University, Kingston, ON K7L3N6, Canada; yeatesk@queensu.ca; 4 School of Global Public Health, New York University, New York, NY 10012, USA; 5 Pamoja Tunaweza Research Centre, Moshi, Tanzania; 6 Perlmutter Cancer Center, NYU Langone Health, New York, NY 10016, USA; Ophira.Ginsburg@nyulangone.org; 7 Division of Cancer Epidemiology and Genetics (DCEG), National Cancer Institutes, Bethesda, MD 20892, USA; maria.demarco@nih.gov (M.D.); schiffmm@mail.nih.gov (M.S.)

**Keywords:** deep learning, cervical cancer, cervix/non-cervix, ensemble, one-class classification

## Abstract

Automated Visual Examination (AVE) is a deep learning algorithm that aims to improve the effectiveness of cervical precancer screening, particularly in low- and medium-resource regions. It was trained on data from a large longitudinal study conducted by the National Cancer Institute (NCI) and has been shown to accurately identify cervices with early stages of cervical neoplasia for clinical evaluation and treatment. The algorithm processes images of the uterine cervix taken with a digital camera and alerts the user if the woman is a candidate for further evaluation. This requires that the algorithm be presented with images of the cervix, which is the object of interest, of acceptable quality, i.e., in sharp focus, with good illumination, without shadows or other occlusions, and showing the entire squamo-columnar transformation zone. Our prior work has addressed some of these constraints to help discard images that do not meet these criteria. In this work, we present a novel algorithm that determines that the image contains the cervix to a sufficient extent. Non-cervix or other inadequate images could lead to suboptimal or wrong results. Manual removal of such images is labor intensive and time-consuming, particularly in working with large retrospective collections acquired with inadequate quality control. In this work, we present a novel ensemble deep learning method to identify cervix images and non-cervix images in a smartphone-acquired cervical image dataset. The ensemble method combined the assessment of three deep learning architectures, RetinaNet, Deep SVDD, and a customized CNN (Convolutional Neural Network), each using a different strategy to arrive at its decision, i.e., object detection, one-class classification, and binary classification. We examined the performance of each individual architecture and an ensemble of all three architectures. An average accuracy and F-1 score of 91.6% and 0.890, respectively, were achieved on a separate test dataset consisting of more than 30,000 smartphone-captured images.

## 1. Introduction

There were nearly 570,000 new cases of cervical cancer diagnosed in 2018 according to the World Health Organization (WHO) [1]. Visual Inspection of the cervix post application of dilute (3–5%) Acetic acid (VIA) is considered to be a simpler and less expensive test compared to the other cervical cancer screening modalities. Cervical tissue whitening after the acetic acid application (called acetowhitening or AW) is an indicator of Human Papillomavirus (HPV) infection. However, not all AW lesions are indicative of cervical intraepithelial neoplasia (CIN), and not all CIN turns white with acetic acid application. This results in relatively poor quality assessments using VIA, where the typical sensitivity and specificity of VIA are in the range from ~70–80% as reported in [2,3,4]; but, high subjectivity has also been reported in [5] with only a 56.8% complete decision agreement rate among 20 gynecologists over 919 cases. A breakthrough deep learning method, called Automated Visual Evaluation (AVE) [6], was shown to successfully classify digitized cervix outperforming human experts on a test dataset especially for a key age group in women. The method was proven on a large high quality collection of over 60,000 digitized images taken with a specialized SLR camera in a 7 year longitudinal study of over 9000 women [7,8]. We have recognized that future images that are likely to be acquired with low cost handheld devices, such as a smartphone, could impact AVE performance if the cervix images are of low quality. In response, we have embarked on a multi-national multi-organization collaborative effort [9] to develop and validate a smartphone-based AVE app with enhanced training on a wide variety of images. We envisage the app to include pre-classification image quality assessment to ensure reliable results.

**Figure 1:**
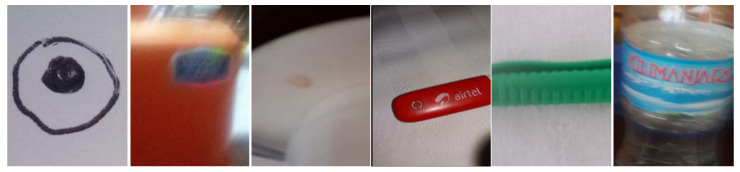
Non-cervix image samples in one collected dataset.

Among various tasks necessary for reliable AVE performance, two have drawn our attention: ensuring that the images used for processing are (i) of adequate quality; and, (ii) the images have adequate coverage of necessary anatomical landmarks, such as the os, the squamo-columnar junction, and the transformation zone. Acceptable image quality can be defined in many ways, namely focus, illumination, occlusion, and content coverage. So far, we have conducted the following peripheral studies in order to support AVE: (i) focus determination: we developed a deep learning algorithm to detect sharp cervical regions in cervix images captured using mobile devices [10]; and, (ii) anatomical landmark detection: we developed a deep learning algorithm to automatically locate and segment the os [11]. However, in these two peripheral studies [10,11], the methods assume that the input images are images of the cervix. However, we have observed in our visual analysis of cervical image datasets, particularly those that are retrospectively collected from patient records receiving routine gynecological care, that they often contain images with insufficient cervical information and/or non-cervix images. Possible reasons could be accidental camera trigger presses, or using the device for training (in which case precise image acquisition is not crucial) among others. Several examples of non-cervix images from a dataset are shown in Figure 1. It is necessary to remove these non-cervix images since they distract model training and could adversely impact classifier performance. Manual inspection during and after the capture of images can be done, but it is labor intensive, distracting during clinical procedures, and prone to human error.

Our goal is to develop an automatic approach which labels images as cervix or non-cervix. This is work toward developing a pre-processing quality control module that would ensure that such images are not associated with study subject data or patient data.

One of the challenges in automatically labeling these images is large visual variety in image content due to the cervices being morphologically different with respect to each individual’s age, parity, and related changes in her anatomy. Other factors creating variability include image focus, illumination, specular reflection from the moist tissue, presence of clinical instruments (such as the speculum and swab), and variable zoom. Secondly, our datasets are highly imbalanced with respect to the numbers of cervix images and non-cervix images with many more cervix images than non-cervix images.

To the best of our knowledge, there is no previously reported study regarding classifying an image to be cervix or non-cervix. We propose using an ensemble of three methods, aiming to take advantage of the strengths of each. They are one-class classification, binary classification, and object detection, namely Deep SVDD [12], a customized CNN (Convolutional Neural Network), and RetinaNet [13].

The rest of the manuscript is organized as follows: Section 2 describes the details of datasets used in this study; Section 3 describes the methods; Section 4 presents the experiments and a discussion of the results, with Section 5 offering conclusions.

## 2. Image Data

Four datasets are used in this study: MobileODT, Kaggle, SEVIA, and COCO2017 [14]. Of these, MobileODT, Kaggle, and SEVIA are cervix image datasets, while the COCO dataset is of stock photography images. We use the MobileODT, Kaggle, and COCO datasets for training and validation, and we use the SEVIA dataset for testing. All datasets are de-identified and exempted from IRB review. Note that training, validation and testing sets have no overlap on any image. All these datasets have large intra- and inter-dataset variety with respect to object illumination, lighting condition, object color, object position, size, and other related visual factors.

### 2.1. MobileODT Dataset

The images in this dataset were provided by MobileODT (https://www.mobileodt.com/) originally collected mainly for the purpose of documenting the colposcopy procedure using their hand-held colposcope (EVA), which incorporates a mobile device for image capture. The dataset contains 9000 images labeled by 6 experts (at Hospital Universitario de Caracas), and verified for completeness by staff at MobileODT and the National Cancer Institute (NCI). The dataset exhibits excellent natural variety in the appearance of women’s cervices due to age, parity, medical history, and ethnicity. We use this image dataset for training deep learning algorithms in the cervix class. We apply a cervix detector used in [10] to detect the cervix regions; we record these regions using a bounding box format as part of the annotation for training the RetinaNet framework used in our study. There are 7984 cervix images in this dataset. All of them are used to train the Deep SVDD and the customized CNN. Of the total dataset, 3118 images have bounding box annotations and are used for training the RetinaNet.

### 2.2. Kaggle Dataset

The dataset on Kaggle [15] provided for the “Intel & MobileODT Cervical Cancer Screening Competition” was originally released for classifying the images into three cervix types based on changes in their visual appearance; these three types are related to the changing, age-related, relationships between squamous and glandular epithelium of the cervix. Cervix region contours for the images in this set are also available [16]. For RetinaNet training, a tight fitting bounding box around these contours is computed and stored. In each image, we obtain the cervix bounding box by drawing a rectangle tightly enclosing the given contour. We observe that the image quality in this set varies widely. We note presence of motion blur, speculum interference, and specular reflection, poor lighting, poor camera positioning, among others. We use the 1481 training images from [15] in our training, and the 512 test images from [15] in validation.

### 2.3. COCO Dataset

**Figure 2:**
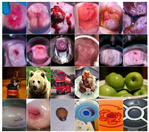
Examples of cervix images used in this study, showing quality variability. 1st row: MobileODT dataset; 2nd row: Kaggle dataset; 3rd row: COCO dataset; 4th row: SEVIA dataset (only the first two images are cervix images, the rest images in this row are non-cervix image samples in this dataset).

COCO [14] is a large-scale dataset collected for the general purpose tasks of object detection, segmentation, captioning, etc. It contains hundreds of thousands of stock photography images categorized into as many as 80 classes, viz., airplane, motorcycle, dog, cat, etc. (example shown as the 3rd row in Figure 2), thereby providing us with a variety of samples with characteristics that are different from cervix images, fulfilling our need of a good resource for non-cervical features in training. We use COCO2017 [14] as the supplement for our non-cervix image dataset in training and validation. Since there is no presence of a cervix in these images, we annotate each image with (i) a sequence containing no coordinate, indicating “absence of RoI” (for RetinaNet); and (ii) the non-cervix class label. The quantitative details of images used in this dataset for training, validation of each method is shown in Table 1.

### 2.4. SEVIA Dataset

This dataset was collected across 4 regions of Tanzania by the “Smartphone-Enhanced program of Visual Inspection with Acetic acid (SEVIA)” for cervical cancer screening in low-resource regions [17]. The program makes the use of smartphone camera as a training tool to allow health providers to maintain and strengthen their skills in VIA. This dataset is suitable for our study in three aspects: (i) it has images in a large quantity (31,967); (ii) it contains non-cervix images. While small percentage-wise, the number of non-cervix images (1816) is still substantial; (iii) large image quality variation can be observed among the cervix images, where the most common degradation is “motion blur”; and; (iv) many of the non-cervix images in this dataset are visually similar to actual cervix images, such as hand-drawn illustration images of cervix (4th row in Figure 2), or have similar visual features with cervix, such as shape, edges, color, etc. Those images were included in the dataset probably for the purpose of medical training. The SEVIA dataset may be considered to be a very challenging image collection source for classifying cervix images/non-cervix images. We use it as the test dataset.

## 3. Methods

In this study, we address the problem of cervix/non-cervix classification from two aspects: (1) building a balanced dataset containing both cervix and non-cervix images, and (2) utilizing multiple deep learning architectures. Regarding the data imbalance issue in Kaggle and MobileODT datasets, we use images in COCO2017 as our non-cervix images in training and validation. The COCO images are randomly selected from 80 categories in general domain, and are labeled as non-cervix images regardless of their original labels in COCO dataset. Together with cervix images from MobileODT and Kaggle datasets we build balanced training and validation datasets. As for the testing dataset, we use the SEVIA dataset which is not seen by any of the models in training. All these datasets have large intra- and inter-dataset variety with respect to object illumination, lighting condition, object color, object position, size, and other related visual factors. The detailed dataset description is given in Table 1.

We propose an ensemble method which consists of three deep learning architectures: RetinaNet, Deep SVDD and a customized CNN. We use RetinaNet since it has been reported to achieve state-of-the-art performance for multi-class object detection on public datasets such as ImageNet and COCO. In addition, we use Deep SVDD which was proposed for anomaly detection [12] known as a “one-class classification” problem [18]. The core of the method is to train a neural network to extract and encode the common factors of training data (mainly consisting of “normal” data) variation into a hypersphere of minimum volume, so that the targeting class data (normal) can be separated from the counter-examples (anomalous) by the hypersphere. The concept suits the scenario in our study where the “anomalous” samples could be of unlimited representations. By obtaining a good “normality description”, we aim to accurately distinguish the “normal” samples, “cervix images”, from the “anomalous” samples, “non-cervix” images. In addition to RetinaNet and Deep SVDD, we propose a binary classifier which is a sequential CNN with a simple architecture. It consists of four sequentially connected convolutional blocks followed by two fully-connected layers. The three methods, RetinaNet, Deep SVDD, and the customized CNN, are different in several aspects such as the algorithm type, the network architecture, and how the class labels are predicted, and are complementary to each other. We fuse their outputs via a voting scheme, making an ensemble method. The ensemble method as well as each individual model is tested on a separate image set that is not used in training and validation. The performance of the ensemble method is compared with that of each model. Limitations of the methods are also discussed through analyzing the error cases in both validation and testing. Below is a brief introduction to each architecture.

### 3.1. RetinaNet

**Figure 3:**
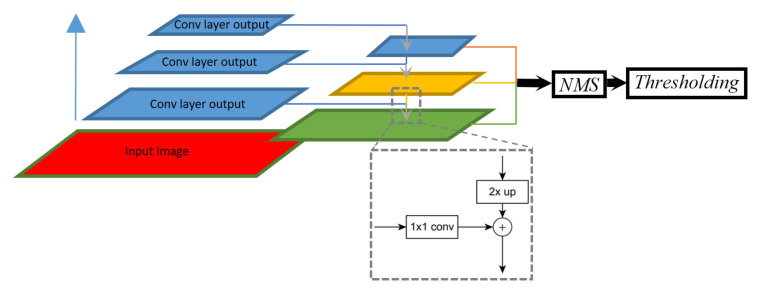
RetinaNet workflow. The convolutional layers (blue block on the left) extract features in different scales (blue, yellow and green layers in the middle) followed by NMS (Non-maximum Suppression) and thresholding.

We use an object detection network, RetinaNet [9], in this study. It has reportedly been achieving state-of-the-art performance for multi-class object detection on public datasets such as ImageNet [19] and COCO [13]. It has a feature pyramid network [20] where features are computed separately in multiple scales and then merged through convolutional operations (top-down pathways) (Figure 3). Furthermore, with the proposed “focal loss” function, RetinaNet is reported to have better performance handling class imbalance and focusing on hard, misclassified examples. To help us achieve a faster convergence on training, we initialize the RetinaNet with pre-trained weights obtained in one of our related studies [10] which also used a subset of the cervix images captured using a mobile device by MobileODT.

As introduced in Section 2.2 and Section 2.3, to prepare annotation for training this supervised algorithm, we (i) annotate cervix images with a class label (“cervix”) and cervix bounding box coordinates; (ii) annotate non-cervix images with a class label (“non-cervix”) and an empty coordinate sequence. Based on the learned information from the annotated region of interest (RoI), the network outputs a score associated with each candidate bounding box. The score indicates the probability (0–1) that the bounding-box-enclosed region is the desired target class. Non-maximum suppression is then applied to merge similar bounding boxes that are with high IoU (Intersection over Union) values. Lastly, a class label is generated by thresholding the score of each bounding box. If there is no bounding box predicted with a score higher than the threshold, that image is considered as a non-cervix image. The threshold is selected based on the performance of the validation dataset; more details are presented in Section 4.

### 3.2. Deep SVDD

**Figure 4:**
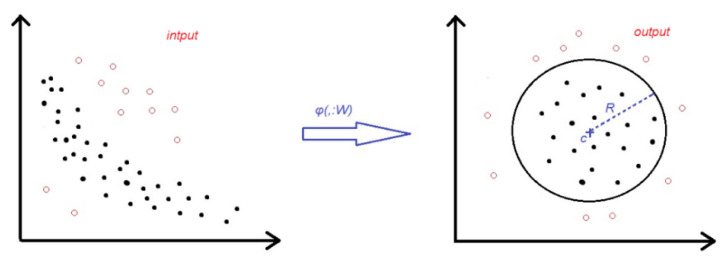
Deep SVDD [21] workflow from input data distribution to output data distribution. The “hypersphere” (the circle in the right subfigure) is found by the algorithm as the smallest hypersphere with center c, and radius R, that includes all (or the majority) of the target samples in the feature space.

The second classifier we use is Deep SVDD [12] which is a one-class classification method built with a deep learning architecture. It learns a deep neural network transformation φ (·; W) (with weights W) that attempts to map most of the target data representations into a “hypersphere” of minimum volume, so that new samples belonging to the target category would be mapped within this hypersphere whereas the samples belonging to the other category would be mapped outside of it (Figure 4). The role of the deep architecture is to extract features from the input images and learn common factors to represent the target category, in our case, the cervix images. This one-class classification concept is a good fit for our study scenario, where the “anomalous” samples could have unlimited representations.

We modify the original Deep SVDD structure in [12] lightly to make it suitable for encoding larger images. The architecture proposed in [12] used LeNet [21]-type CNNs for the datasets of MNIST and CIFAR-10. Most images in these datasets have a size of 32 × 32, which is much smaller than the size of our cellphone images. To make the structure more suitable for encoding images as large as 128 × 128, we modify the network structure by increasing the number of units in the final dense layer. The architecture has three convolutional modules with 32 × 5 × 5 filters, 64 × 5 × 5 filters, and 128 × 5 × 5 filters, and a final dense layer of 256 units.

### 3.3. Customized CNN

**Figure 5:**
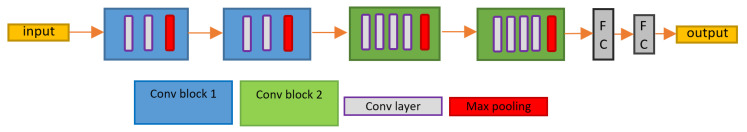
Customized CNN (Convolutional Neural Network) architecture.

Compared with the above two deep learning architectures, viz., Deep SVDD and RetinaNet, our Customized CNN is conceptually and architecturally simpler. Following similar architectures of classic CNNs such as VGG and LeNet, we design the CNN as a sequential architecture (Figure 5) that consists of 4 convolutional blocks and two fully-connected layers. In each of the four convolutional blocks, ReLU (Rectified Linear Unit) activation is used for every convolutional layer and a max pooling layer is connected to the last convolutional layer in each block. The first two blocks (“Conv block 1” in Figure 5) consist of 2 convolutional layers each, and the last two blocks (“Conv block 2” in Figure 5) consist of 4 convolutional layers each. Two fully-connected layers consist of 64 nodes and 2 nodes, respectively. Softmax activation is used for the last fully-connected layer. The loss function is categorical entropy loss and the optimization is performed via Stochastic Gradient Decent (SGD).

### 3.4. Ensemble Method

The output scores of each individual network are obtained through different mechanisms. RetinaNet uses sigmoid activation at the output, our customized CNN uses the softmax function, and both limit the output scores to be less than 1. However, Deep SVDD uses CNN architecture(s) to encode the input and find the “hypersphere (radius)” for distinguishing samples belonging to different categories. The output of Deep SVDD represents the “encoded distance” of the given sample to the center of the hypersphere which is not limited. To combine outputs from the above three methods, we take advantage of a voting method using the predicted labels (a cervix image or non-cervix image label predicted by each method) instead of using the score values. The class label which is “voted” at least twice is assigned to the input image as the final decision of the ensemble method.

## 4. Experiments

### 4.1. Data Preparation and Implementation Details

Table 1 summarizes the data splits used in this work. Note that the number of validation images in MobileODT or Kaggle datasets are the same for all three methods. The total number of validation images are adjusted by the amount of COCO images used, with respect to the ratio of the number of cervix images over the number of non-cervix images in training.

The test dataset contains 31,967 (30,151 cervix images + 1816 non-cervix images) images, which has significant imbalance between the two categories. Due to this imbalance, we split the cervix images and generate a “10-fold” testing scenario. In each fold, we have: (i) all the non-cervix images (1816 images); and (ii) 3015 cervix images that are randomly selected (the last fold has 3016 images). Note that there is no image that is selected more than one time; in other words, for the cervix images, there is no overlap between each test fold. The purpose of splitting the whole set of cervix images into many folds is to maintain relatively balanced test sets, so that in each test, the performance measurement parameters we use, such as accuracy, sensitivity (Sens.), specificity (Spec.) and F-1 scores are calculated without bias brought by dataset imbalance [22]. We then use the average value of those measures to evaluate the performance of each individual algorithm as well as the ensemble model. Each image is resized to three sizes and the input image size for each network architecture is listed in Table 2.

The training is undertaken with one Nvidia Tesla V100 graphical processing unit (GPU). The hyper-parameters for each model are:

- RetinaNet: The batch size is 4, and a learning rate starting at 0.001. The weight decay is set as 0.0001 and the momentum is 0.9. The model uses ResNet50 [23] as the backbone and is initialized with pre-trained RetinaNet weights that were obtained in [10]. Augmentations of rotation, shearing, shifting and x/y flipping are used. Python 3.6 and Keras 2.2.4 are used.
- Deep SVDD: The batch size is 8 and the weight decay factor of λ = 0.5 × 10^−5^. Base learning rate is set as 0.001. Python version is 3.7 and PyTorch version is 0.5. Our Deep SVDD network is modified based on the PyTorch implementation in [24].
- Customized CNN: The batch size is 16. Learning rate is set as 10^−4^ and momentum is set as 0.9. “Categorical cross entropy” loss is used and updated via Stochastic Gradient Descent (SGD). Augmentations of scaling, horizontal/vertical shift and rotation are used. Python 3.6 and Keras 2.2.4 are used.

### 4.2. Results and Disccussion

The performance of each individual method is evaluated first followed by the performance of the ensemble model.

#### 4.2.1. Results of RetinaNet

**Figure 6:**
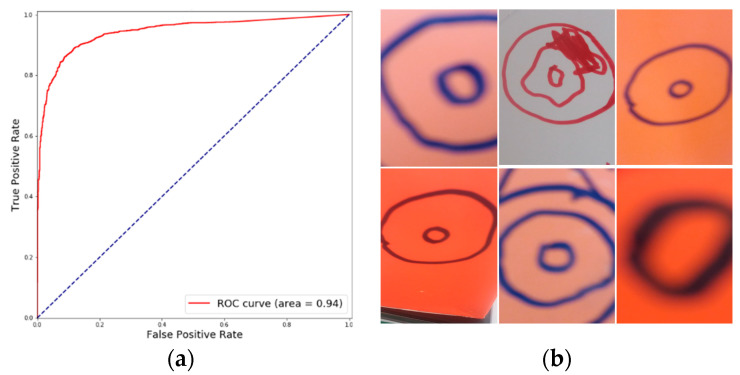
RetinaNet Results: (**a**) ROC from one of the test folds, and (**b**) and false negative error examples.

The performance of the RetinaNet model on the test sets is shown in Table 3a,b. It achieves an average accuracy of 91.1%, an average F-1 score of 0.877 and AUC of 0.94 (Figure 6a). From the error examples shown in Figure 6b, we can observe that the error cases (mainly false negatives) are hand-drawn sketches of the cervix. These misclassifications are due to their visual features that are similar with cervical features, e.g., edges, colors, etc.

#### 4.2.2. Results of Deep SVDD

**Figure 7:**
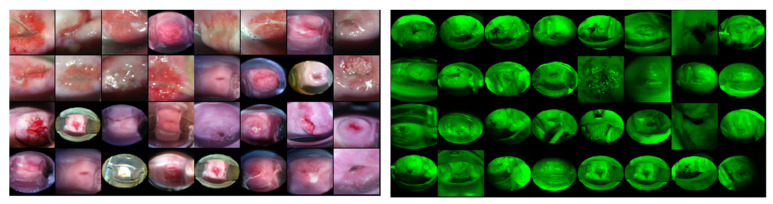
Validation examples of Deep SVDD approach. Left images are predicted as cervix in the validation set. Right images predicted as non-cervix images, though all of the images shown are cervix image samples with “green filter” applied.

As shown in Table 4a,b, our modified Deep SVDD achieves an average F-1 score of 0.863 across the folds, and the “most normal” samples are shown in Figure 7.

We observe that there are some green-filtered cervix images that are from the Kaggle dataset [15] in the training/validation sets, as shown in Figure 7. There are no such green filtered images in the SEVIA dataset. It is worthwhile to point out, however, that they are predicted with large “anomaly scores” [12] in the Kaggle validation set. This indicates that there is a significant visual difference from the majority of “cervix image” samples used to train Deep SVDD. On further examination, we find that the number of training images with a green filter is less than 0.1% of all training cervix images, which may be insufficient for the model to learn as an acceptable appearance for cervix images.

#### 4.2.3. Results of Customized CNN

As shown in Table 5a,b, the customized CNN achieves an average accuracy of 83.0% on the test folds. The overall performance for classifying the cervix image class is lower than that of the previous two methods, viz., RetinaNet and Deep SVDD. However, it obtains an accuracy over 93% on the non-cervix images in the validation set, with a threshold value of 0.75. We use this parameter setting in the test phase.

#### 4.2.4. Results of Ensemble Method

We obtain the hyper parameters for each of above three methods from their best validation performance and use the parameters in the ensemble methods in the testing stage. Given an input testing image, a label of cervix or non-cervix is assigned if that specific label is voted for at least twice. As shown in Table 6a,b, we have achieved an average testing accuracy of 91.6% with F-1 score of 0.890 for the ensemble method.

**Figure 8:**
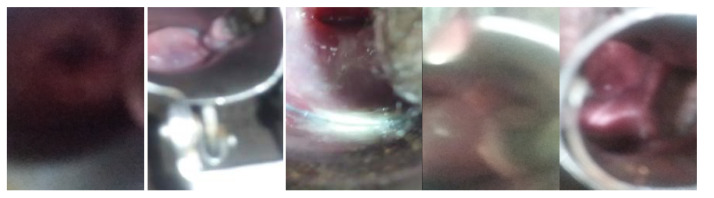
Samples of cervix images that are “misclassified” as non-cervix images.

The specificity (0.900) achieved by the ensemble method is very close to that of RetinaNet which yields the best specificity (0.908) among the three individual methods. A higher sensitivity (0.935) is achieved by the ensemble method indicating an improvement in classifying non-cervix images over the individual methods. Compared with each individual method, the ensemble method detects more non-cervix images at the cost of “losing” a small amount of cervix images. Meanwhile, we observe that many error cases here show similar visual characteristics to those found in each individual model. Among the cervix images that are misclassified as non-cervix images (false positive), there are often factors such as motion blur, low lighting, presence of distractors such as pubic hair, that reduce the visual clarity of the cervix as well as the sharpness of the entire image (shown in Figure 8). While, in reality, these are cervix images, they would not be typically used in visual analysis by human experts due to poor quality. While our results are “wrong”, the ability of our ensemble method to “reject” these images is advantageous for quality control of cervix image acquisition and use in machine learning algorithms.

## 5. Conclusions

Toward our goal of achieving reliable AVE performance, it is critical that the algorithm be presented with images (1) that contain the cervix; and (2) are of adequate quality. In this paper, we have presented an ensemble deep learning method which detects non-cervix images so that they can be eliminated from AVE processing. The ensemble structure consists of three deep learning methods, viz., RetinaNet, Deep SVDD, and a customized CNN, that are selected to take advantage of their complementary features, viz., object detection, one-class classification, and binary classification. The decisions of these three models are combined via a voting scheme. 

The individual methods as well as the ensemble decisions are evaluated against a large smartphone-acquired cervix image dataset that has many non-cervix images. The results from our testing show that the ensemble method outperforms individual deep learning methods and achieves an accuracy and F-1 score of 91.6% and 0.890, respectively. While this performance is respectable, we analyzed the error cases to better understand why the ensemble was wrong. We observe that low image quality factors, such as motion blur, poor illumination, and incomplete/occluded cervix view (shown in Figure 8) among others, are responsible for these errors. As a result, a more comprehensive image quality and cervix/non-cervix classifier is necessary to ensure that only the most appropriate images are presented to the AVE. There are other errors more difficult to resolve. Note the sketches of the cervix that were possibly used for training (shown in Figure 6b) caused false negatives, i.e., non-cervix images classified as a cervix. This could be addressed by adding such examples to the training dataset. 

Our approach can help the cervix image acquisition in two usage scenarios. The method can be used either as a standalone module to process all the images that have been uploaded to a data server or an embedded module in an image capturing device to process each image shortly after it is captured. The cervix images identified by the classifier can be sent back to human experts for further review, or to another automatic algorithm.

We believe that such data quality algorithms are extremely important for not only cleaning “big data” datasets used in developing robust disease detection algorithms, but also for quality assurance for machine learning algorithms in routine clinical use.

## Figures and Tables

**Table 1:** Summary of data split and quantitative details of images used from each dataset.

Dataset	Training			Validation	Testing
	RetinaNet	Deep SVDD	Custom CNN	All Methods	All Methods
MobileODT	3118	3118	7170	814	0
Kaggle	1481	1481	1481	512	0
COCO	4599	0	8561 (contains the 4599 images used for training RetinaNet)	1326	0
SEVIA	0	0	0	0	30,151 + 1816 (in 10 folds, last fold has 3016 cervix images)

**Table 2:** Input image dimensions for all the three architectures used.

Methods	Resized Dimension (w × h)
Deep SVDD	128 × 128
RetinaNet	480 × 640
Customized CNN	256 × 256

**Table 3:** Performance measurement (**a**) and the confusion matrix of one test fold (**b**) for RetinaNet approach on test images. Positive: non-cervix image. (Sens. = Sensitivity, Spec. = Specificity, Std. = Standard deviation, Avg. = Average).

(**a**)					
**Methods**	**Accuracy (Avg./Std.)**	**Sens. (Avg.)**	**Spec. (Avg./Std.)**		**F-1 Score (Avg.)**
RetinaNet	0.911/0.010	0.918	0.908/0.011		0.877
(**b**)					
		**True Positive**		**True Negative**	
**Predicted Positive**		1667		279	
**Predicted Negative**		149		2736	

**Table 4:** Performance measurement (**a**) and the confusion matrix of one test fold (**b**) for Deep SVDD approach on test images. Positive: non-cervix image. (Sens. = Sensitivity, Spec. = Specificity, Std. = Standard deviation, Avg. = Average).

(**a**)					
**Methods**	**Accuracy (Avg./Std.)**	**Sens. (Avg.)**	**Spec. (Avg./Std.)**		**F-1 Score (Avg.)**
Deep SVDD	0.889/0.010	0.922	0.876/0.010		0.863
(**b**)					
		**True Positive**		**True Negative**	
**Predicted Positive**		1674		394	
**Predicted Negative**		142		2621	

**Table 5:** Performance measurement (**a**) and the confusion matrix of one test fold (**b**) for customized CNN approach on test images. Positive: non-cervix image. (Sens. = Sensitivity, Spec. = Specificity, Std. = Standard deviation, Avg. = Average).

(**a**)					
**Methods**	**Accuracy (Avg./Std.)**	**Sens. (Avg.)**	**Spec. (Avg./Std.)**		**F-1 Score (Avg.)**
Customized CNN	0.830/0.010	0.924	0.780/0.012		0.810
(**b**)					
		**True Positive**		**True Negative**	
**Predicted Positive**		1678		667	
**Predicted Negative**		138		2348	

**Table 6:** Performance measurement (**a**) and the confusion matrix of one test fold (**b**) for ensemble approach on test images. Positive: non-cervix image. (Sens. = Sensitivity, Spec. = Specificity, Std. = Standard deviation, Avg. = Average).

(**a**)					
**Method**	**Accuracy (Avg./Std.)**	**Sens. (Avg.)**	**Spec. (Avg./Std.)**		**F-1 Score (Avg.)**
Ensemble	0.916/0.004	0.935	0.900/0.008		0.890
(**b**)					
		**True Positive**		**True Negative**	
**Predicted Positive**		1698		301	
**Predicted Negative**		118		2715	

## References

[1] Human Papillomavirus (HPV) and Cervical Cancer. https://www.who.int/en/news-room/fact-sheets/detail/human-papillomavirus-.

[2] Bhattacharyya A., Nath J., Deka H. (2015). Comparative study between pap smear and visual inspection with acetic acid (via) in screening of CIN and early cervical cancer. J. Mid Life Health.

[3] Egede J., Ajah L., Ibekwe P., Agwu U., Nwizu E., Iyare F. (2018). Comparison of the accuracy of papanicolaou test cytology, Visual Inspection with Acetic acid, and Visual Inspection with Lugol Iodine in screening for cervical neoplasia in southeast Nigeria. J. Glob. Oncol..

[4] Sritipsukho P., Thaweekul Y. (2010). Accuracy of visual inspection with acetic acid (VIA) for cervical cancer screening: A systematic review. J. Med. Assoc. Thai..

[5] Jeronimo J., Massad L.S., Castle P.E., Wacholder S., Schiffman M., National Institutes of Health (NIH)-American Society for Colposcopy and Cervical Pathology (ASCCP) Research Group (2007). Interobserver agreement in the evaluation of digitized cervical images. Obstet. Gynecol..

[6] Hu L., Bell D., Antani S., Xue Z., Yu K., Horning M.P., Gachuhi N., Wilson B., Jaiswal M.S., Befano B. (2019). An observational study of deep learning and automated evaluation of cervical images for cancer screening. J. Natl. Cancer Inst..

[7] Bratti M.C., Rodriguez A.C., Schiffman M., Hildesheim A., Morales J., Alfaro M., Guillén D., Hutchinson M., Sherman M.E., Eklund C. (2004). Description of a seven-year prospective study of human papillomavirus infection and cervical neoplasia among 10000 women in Guanacaste, Costa Rica. Rev. Panam. Salud Publica.

[8] Herrero R., Schiffman M., Bratti C., Hildesheim A., Balmaceda I., Sherman M.E., Greenberg M., Cárdenas F., Gómez V., Helgesen K. (1997). Design and methods of a population-based natural history study of cervical neoplasia in a rural province of Costa Rica: The Guanacaste Project. Rev. Panam. Salud Publica.

[9] AI Approach Outperformed Human Experts in Identifying Cervical Precancer. https://www.cancer.gov/news-events/press-releases/2019/deep-learning-cervical-cancer-screening.

[10] Guo P., Singh S., Xue Z., Long L.R., Antani S. Deep learning for assessing image focus for automated cervical cancer screening. Proceedings of the 2019 IEEE EMBS International Conference on Biomedical & Health Informatics (BHI).

[11] Guo P., Xue Z., Long L.R., Antani S. (2020). Deep learning cervix anatomical landmark segmentation and evaluation. SPIE Med. Imaging.

[12] Ruff L., Vandermeulen R.A., Geornitz N., Deecke L., Siddiqui S.A., Binder A., Müller E., Kloft M. Deep one-class classification. Proceedings of the 35th International Conference on Machine Learning, PMLR.

[13] Lin T.Y., Goyal P., Girshick R., He K., Dollar P. (2017). Focal loss for dense object detection. ICCV.

[14] Common Objects in Context (COCO) Dataset. http://cocodataset.org/#home.

[15] Intel & MobileODT Cervical Cancer Screening Competition, March 2017. https://www.kaggle.com/c/intel-mobileodt-cervical-cancer-screening.

[16] Fernandes K., Cardoso J.S. Ordinal image segmentation using deep neural networks. Proceedings of the International Joint Conference on Neural Networks.

[17] Yeates K., Sleeth J., Hopman W., Ginsburg O., Heus K., Andrews L., Giattas M.R., Yuma S., Macheku G., Msuya A. (2016). Evaluation of a Smartphone-Based Training Strategy among Health Care Workers Screening for Cervical Cancer in Northern Tanzania: The Kilimanjaro Method. J. Glob. Oncol..

[18] Moya M.M., Koch M.W., Hostetler L.D. (1993). One-class classifier networks for target recognition applications. Proceedings World Congress on Neural Networks.

[19] Deng J., Dong W., Socher R., Li L.J., Li K., Fei-Fei L. ImageNet: A large-scale hierarchical image database. Proceedings of the 2009 IEEE Conference on Computer Vision and Pattern Recognition.

[20] Lin T.Y., Dollar P., Girshick R., He K., Hariharan B., Belongie S. Feature pyramid networks for object detection. Proceedings of the 2017 IEEE Conference on Computer Vision and Pattern Recognition (CVPR).

[21] Lecun Y., Bottou L., Bengio Y., Haffner P. (1998). Gradient-based learning applied to document recognition. Proc. IEEE.

[22] Johnson J.M., Khoshgoftaar T.M. (2019). Survey on deep learning with class imbalance. J. Big Data.

[23] He K., Zhang X., Ren S., Sun J. (2016). Deep residual learning for image recognition. CVPR.

[24] A PyTorch Implementation of the Deep SVDD Anomaly Detection Method. https://github.com/lukasruff/Deep-SVDD-PyTorch.

